# Using Attainment of the Designated Aquatic Life Use to Determine Adverse Environmental Impact

**DOI:** 10.1100/tsw.2002.296

**Published:** 2002-05-11

**Authors:** Greg Seegert

**Affiliations:** EA Engineering, Science, and Technology, 444 Lake Cook Rd., Suite 18, Deerfield, IL 60015, USA

**Keywords:** adverse environmental impact, 316(b) demonstration, Clean Water Act, impact assessment methods, biological models, conditional mortality rate

## Abstract

Section 316(b) of the Clean Water Act requires that cooling-water intake structures (CWIS) use Best Technology Available (BTA) to minimize adverse environmental impacts (AEI). The U.S. EPA has not defined AEI, and there is no clear consensus regarding its definition. Nonetheless, operational definitions are necessary to evaluate design alternatives and to measure the success of mitigative measures. Rather than having to develop measures of aquatic health that are highly site-specific, controversial, and often unlikely to elicit agreement from all sides of the environmental “fence”, it may be more productive to use existing ecological assessment tools. Aquatic Life Uses (ALU) already provide a regulatory framework to assess the quality (health) of the aquatic community in various habitats (e.g., warmwater habitat, exceptional warmwater habitat). Attainment of the ALU indicates that further point source controls are unnecessary, whereas nonattainment indicates that those pollutants or stressors causing the nonattainment must be reduced. A similar approach for existing water intakes is recommended. That is, attainment of the designated ALU will be taken as an indication that there is no AEI. Although attainment of the ALU may not be a foolproof indicator of a lack of AEI, this approach seems more reasonable that using scarce monetary resources to fix problems that likely do not exist, or having both regulators and the regulated community expend their resources debating whether various observed biological responses do or do not constitute AEI.

## Keywords adverse environmental impact, 316(b) demonstration, Clean Water Act, impact assessment methods, biological models, conditional mortality rate

## Domains freshwater systems, ecosystems and communities, water science and technology, environmental management and policy, environmental technology, environmental monitoring

## INTRODUCTION

In 1972 the U.S. Congress passed Public Law (PL) 92-500, the Federal Water Pollution Control Act Amendments of 1972 (the "Act" or "CWA"). Section 316(b) of the Act addressed cooling-water intake structures (CWIS). This section required that "… the location, design, construction, and capacity of cooling water intake structures reflect the best technology available for minimizing adverse environmental impact (AEI)." Despite the passage of nearly 30 years, scientists, regulators, and resource managers have yet to reach a consensus on what constitutes AEI[1]. This lack of consensus is reflected in the title of this Symposium, "Defining and Assessing Adverse Environmental Impact." Based on the titles of their papers, more than half the authors at this symposium attempted to define what level of organization (individual, population, or community) was appropriate to assess AEI or how AEI should be measured at the chosen organizational level. Thus, in their titles there are words or phrases like "defining and assessing AEI"[2], "indicators of AEI"[3], "the challenge of defining endpoints"[4], and "defining AEI"[5].

As part of a 1995 Consent Agreement, the U.S. EPA is revising Section 316(b) of the CWA. In the final rule for New Sources intakes, signed 9 November 2001, the EPA did not define AEI. Because no definition has been established, the debate over AEI continues, in part because the context for such a definition involves societal as well as scientific considerations[6]. Indeed, one of the more contentious debates during public meetings held in conjunction with promulgating the new 316(b) rules has been how to determine what level of organization to assess. One faction believes that in a societal context, the loss of even one individual (the “one dead fish” position) is unacceptable, while many scientists believe that assessment must be at a higher level of organization (population or community). Given the chasm that exists between these two positions, the EPA will have to display Solomon-like wisdom to appease all parties. Interpretation of AEI is particularly important to the electric utility industry because of the volume of cooling water they use and, at least, the potential to affect fish populations adversely.

Historically, the EPA consistently made decisions regarding 316(b) compliance on a site-specific basis, relying on information provided as part of 316(b) “Demonstrations.”

However, for new sources, the EPA has recently established generic guidelines that apply regardless of the waterbody type (e.g., rivers and streams vs. lakes and reservoirs) on which the CWIS is located. This generic approach makes it even more difficult to define AEI since populations and communities vary from site to site. A final complication is that roughly half of the U.S.'s electric generation capacity is located on large rivers (unpublished EPRI data), but the EPA has no guidance document describing how to assess biological populations or communities in large rivers. Because of this lack of guidance, the EPA has decided against using biological criteria as part of the 316(b) assessment process. Thus, even if agreement is reached on what constitutes AEI, there are currently no standard protocols to measure and assess endpoints on the waterbody type that contains the highest percentage of the nation's generating capacity.

## AQUATIC LIFE USES: A PROPOSED ALTERNATIVE

Rather than continuing the 30-year old debate over what constitutes AEI, or how, when, or where to measure it, I recommend an alternative approach, which is determining whether or not the Aquatic Life Use (ALU) for the waterbody in question has or has not been attained. Water-quality criteria consist of two parts, use designations and criteria to protect those uses. Examples of use designations include aquatic life, public water supply, agricultural water supply, and several others. However, ALU is the only use relevant to assessing AEI.

The ALU designation can be subdivided according to the type (e.g., warmwater, coldwater, seasonal salmonid, etc.) or the quality (general, limited, or modified) of the fishery. Some states have a limited number of ALU categories. For example, Illinois classifies almost all of its waterbodies as General Use, except for a few classified as secondary contact. However, other states have a more elaborate system that considers both the type and quality of the fishery. For example, Ohio classifies streams as warmwater or coldwater. The warmwater streams are further subdivided depending on the quality of the biota and various physical (habitat) constraints. Thus, the ALU in Ohio streams can range from limited-resource water (the poorest quality streams), to modified warmwater habitat, to warmwater habitat, and ultimately to exceptional warmwater habitat, the highest quality streams in the state. If habitat quality imposes certain long-term constraints on the quality of the biota due to factors such as channelization, impoundments, etc., then the stream may be given a "modified" classification, as Ohio has done.

Ohio relies exclusively on biological endpoints to determine whether or not each waterbody is in attainment with regard to its designated ALU. Section 3745-1-07(A)(5) of the Ohio Water Quality Standards indicates that biological criteria established by the state provide "direct measure of the attainment of the … aquatic life uses." This same section goes on to indicate that "demonstrated nonattainment of the applicable biological criteria … will cause the director to seek and establish, if possible, the cause of the nonattainment of the designated use." Lastly, it indicates that (given nonattainment), "the director shall, whenever possible and reasonable, implement regulatory controls or make other recommendations regarding water resource management to restore the designated use." This evaluation is used by the Ohio EPA during each NPDES permit renewal cycle to determine whether or not more stringent effluent limits are needed. If the area is found to be in full attainment of the applicable ALU, then additional point source controls are not recommended. However, if biological scores are low and the area is not in attainment, then further controls over point source discharges are typically required (assuming that the nonattainment is likely due to a water quality problem).

I recommend a similar approach be taken with regard to determination of AEI. There are two basic scenarios:

1. The waterbody containing the CWIS is in attainment of its ALU.
2. The waterbody is not in attainment.

If the waterbody is in attainment of the ALU (Scenario 1), then it should be assumed that there is no AEI, because if there was an AEI, attainment would be precluded. If the waterbody is not in attainment, then an AEI is possible, though not confirmed (Scenario 2). For this approach to work, mechanisms need to be in place for measuring attainment. As discussed later in this paper, these mechanisms are well established in some states but poorly defined in others. A lack of attainment provides empirical evidence that some kind of problem exists but does not establish causation[7]. The lack of attainment could be the result of AEI from a CWIS, but it also could be the result of poor water quality, habitat limitations, or site-specific considerations (e.g., drought conditions). Further studies would likely be needed to establish the cause(s) of the nonattainment. Several recent papers[8,9] address the problem of dealing with multiple stressors. If it can be established that the lack of attainment is due to the CWIS, then mitigative measures to reduce or eliminate the AEI would be required. In this regard, it is interesting to note that even though states are required to list the reason(s) for nonattainment in their biannual report to Congress, losses of aquatic organisms at CWIS have never been identified as a reason for nonattainment.

There are several advantages to the proposed approach. First, as part of Section 305(b) of the CWA, states are already required to determine which of their waterbodies are impaired (i.e., not in attainment). Second, it allows the EPA and the states to focus on those waterbodies that need help (i.e., are not in attainment) and not expend scarce resources on waterbodies that are already meeting their CWA goals (i.e., they are in attainment). Third, owners of CWIS can focus their resources on sites that really need it. If a site containing a CWIS is found to be in nonattainment, the owner of that CWIS may want to initiate a 316(b) demonstration to determine to what extent, if any, the CWIS is contributing to the nonattainment. Simultaneously or alternatively, the owner might choose to investigate other reasons for the nonattainment. If it is found that the nonattainment was entirely or partially caused by the CWIS, then it would be reasonable to expect the owner of the CWIS to take actions to mitigate the AEI (i.e., restore the designated use) as required by the 316(b) rule. Thus, the process follows a logical progression; (a) determine if there is a problem, (b) determine if the CWIS causes or contributes to the problem, and (c) if the CWIS causes or contributes to the problem, then the AEI would be established and mitigation of that AEI would be required. A major benefit of this approach is that is avoids the contentious debate over what constitutes AEI.

Another broad benefit of the approach recommended herein is that it provides flexibility. A state or tribe can establish a variety of ALUs, each with a unique series of indicators and benchmarks. Of necessity, these measures will be biological in nature, but the state could choose those that they believe are most appropriate (fish, benthos, shellfish, or some combination of these) for their particular waterbodies. They would also be free to choose the organizational level they wish to measure. For example, they could look at the individual level (e.g., external anomalies, condition, etc.), at the population level (e.g., the abundance of target species), or at the community level (e.g., multimetric indices like the Index of Biotic Integrity[10,11] or the Invertebrate Community Index[12]). Further flexibility can be gained by varying the benchmark(s) over some spatial scale (e.g., by ecoregion, river basin, or even waterbody-specific).

## A CASE HISTORY EXAMPLE

To illustrate how this approach would work, biological data from two power plant sites on the Muskingum River in Ohio are discussed below. The Ohio EPA[12] uses three biological indices to determine attainment of ALU, the Index of Biotic Integrity (IBI)[10,11], the Index of Well-Being (IWBmod) (modified from Gammon[13]), and their own Invertebrate Community Index[12]. Recent benthic data are not available for the Muskingum River sites so this example will focus on the two fish indices. For this region of Ohio, the Ohio EPA has established biocriterion of 40 for the IBI and 8.6 for the IWBmod for collections made by boat electrofishing.

In 1999, from late June through September, boat electrofishing collections were made near two power plants on the Muskingum River, the Muskingum River Plant (MRP), and the Conesville Generating Station (CGS). Five stations were established near each plant, two upstream of the plant and three downstream of it. All collections were made following standard Ohio EPA protocols[12]. At MRP, the mean IBI score both upstream and downstream of the plant was 40. The mean IWBmod score upstream of the plant was 8.9, nearly identical to the score (8.8) downstream of it. Because mean index scores all met the respective biocriterion for this river, the logical conclusion is that the CWIS at the MRP does not cause AEI. This is not to say that the CWIS had absolutely no effect as it surely entrained and impinged a certain number of fish. However, the fact that the area continues to meet objectively established biocriteria and thus is in attainment of the designated use indicates that these effects are biologically insignificant, and thus do not constitute AEI.

Although this example was for an operating CWIS, this process could also be used for new sources. Baseline studies could be conducted before the CWIS was operational. If monitoring after the CWIS began operating demonstrated that the area was no longer in attainment, a logical conclusion would be that the subsequent lack of attainment was due to the CWIS (assuming other factors remained the same during the pre- and post-operational periods).

The situation at the CGS is somewhat more complex. The Ohio EPA has studied this reach of the river several times, most recently in the mid-90s[14]. Their studies suggested that several stressors were, or at least had been, present. Thermal stress was identified as a problem in 1988 during extremely low flows. However, thermal load management by the CGS appears to have addressed this problem. Organic loadings from an upstream paper mill appear to be the most significant current problem[14]. During the 1999 studies, mean IWBmod scores upstream and downstream of CGS were similar (8.8 and 8.5, respectively) and both met the biocriterion of 8.6 (the Ohio EPA allows a deviation below the criterion of 0.5 IWB units to account for normal variability). IBI scores both upstream and downstream of the plant (44 and 37, respectively) met the biocriterion of 40 (for the IBI, the Ohio EPA allows a deviation of 4 units). However, the difference in mean scores between the two areas was statistically significant. The reasons for the upstream/downstream difference are unclear but did not appear to be related to the CWIS. This difference might have been attributable to the thermal component of the discharge, to a DO sag associated with the upstream paper mill, or other factors. Given the facts that (1) both areas meet the biocriterion, and (2) this spatial difference was likely not attributable to the CWIS, it is reasonable to conclude that there is no intake-related AEI at CGS.

Thus, studies near two large CWIS on the Muskingum River demonstrate that measuring attainment of the designed ALU is appropriate and workable, as well as a reasonable surrogate to establish AEI.

## LIMITATIONS OF ALU APPROACH

According to Section 305(b) of the CWA, states are required to determine which waterbodies within their jurisdiction are in attainment and which are not (i.e., which are impaired). Theoretically, each state should already have in place objective quantifiable mechanisms for determining attainment/nonattainment. Some states (e.g., Ohio) do have such procedures in place and in the case of Ohio have even codified the process. However, in other states, the process is less defined and more subjective, thereby making the assessment process more difficult. In these situations, more refined ALUs and/or more detailed guidance regarding the process of how attainment is determined may be necessary. Although it is the responsibility of the states, tribes, and EPA to develop such guidance, the burden of these development efforts may fall to 316(b) applicants. Because, however, the states are already required to address the issue of attainment/nonattainment, the process recommended herein should add little to the regulatory burden of each state.

It takes advantage of something the states already have to do. Furthermore, following this approach will side step the contentious issue of what constitutes AEI, and it will allow states to focus on those waterbodies where there are demonstrated problems.

## ACKNOWLEDGMENTS

The author would like to thank Dr. Tim Lohner and Mr. Chris Yoder for helpful comments they provided on a draft of this paper.
